# Generation of tools for expression and purification of the phage-encoded Type I restriction enzyme inhibitor, Ocr

**DOI:** 10.1099/mic.0.001465

**Published:** 2024-07-23

**Authors:** Joana Alves, Inga Dry, John H. White, David T.F. Dryden, Nicola N. Lynskey

**Affiliations:** 1 The Roslin Institute, University of Edinburgh, Easter Bush Campus, Midlothian, Scotland, EH25 9RG, UK; 2 EaStCHEM School of Chemistry, University of Edinburgh, The King’s Buildings, Edinburgh, EH9 3FJ, UK; 3 Department of Biosciences, University of Durham, South Road, DH1 3LE, UK

## Abstract

DNA manipulation is an essential tool in molecular microbiology research that is dependent on the ability of bacteria to take up and preserve foreign DNA by horizontal gene transfer. This process can be significantly impaired by the activity of bacterial restriction modification systems; bacterial operons comprising paired enzymatic activities that protectively methylate host DNA, while cleaving incoming unmodified foreign DNA. Ocr is a phage-encoded protein that inhibits Type I restriction modification systems, the addition of which significantly improves bacterial transformation efficiency. We recently established an improved and highly efficient transformation protocol for the important human pathogen group A *Streptococcus* using commercially available recombinant Ocr protein, manufacture of which has since been discontinued. In order to ensure the continued availability of Ocr protein within the research community, we have generated tools and methods for in-house Ocr production and validated the activity of the purified recombinant protein.

**Impact Statement**

The generation of bacterial mutants is integral to the continued advancement of research into bacterial physiology and pathogenesis. Such genetic manipulation requires the successful uptake and maintenance of exogenous DNA, a process hindered by natural resistance mechanisms including restriction modification systems that are widespread amongst bacterial pathogens. While deletion of these systems is frequently associated with improved DNA uptake and retention, this is a challenging process that also has the potential to impact diverse biological processes, and limits mutagenesis to only a small number of strains, excluding important clinical isolates. We have thus developed a protocol to transiently block the activity of Type I restriction modification systems, using the phage protein Ocr, that significantly improves bacterial uptake of exogenous DNA. Here we provide the details of generation of an expression plasmid pET19b-ocr, and a protocol for in-house expression and purification of recombinant Ocr protein, with the aim of enabling the improved efficiency of genetic manipulation of multiple bacterial pathogens at low cost and with no impact on bacterial behaviour.

## Data Summary

Methodology for expression and purification of recombinant Ocr protein.

Validation of activity of in-house purified recombinant Ocr protein.

The authors confirm all supporting data, code and protocols have been provided within the article or through supplementary data files.

## Introduction

The development of new tools to facilitate the genetic manipulation of bacterial pathogens for the generation of clean mutants and large mutant libraries has been central to our improved understanding of bacterial physiology and virulence [1,2]. Despite significant progress, research on certain species and/or lineages remains challenging due to their inherent limited genetic tractability [3,5]. Blockade of genetic flux is largely attributed to specific defence mechanisms that have evolved to protect the bacteria from natural acquisition of exogenous and potentially harmful DNA [6,8]. Of these mechanisms, the widely dispersed bacterial restriction modification systems (RMS) have frequently been associated with poor genetic tractability [8,10].

RMS facilitate the discrimination between self and non-self DNA, and specifically target non-self DNA for cleavage and degradation [11,12]. This is achieved by sequence-specific methyl-transferase activity, that protects the host bacterial DNA from cleavage by cognate restriction endonuclease activity targeting the same DNA sequence [11,12]. Thus, RMS activity can significantly impair the genetic tractability of strains when incoming DNA lacks a specific methylation signature, and has limited the repertoire and diversity of clinically important species that can be genetically modified. A number of solutions including the generation of passaging *E. coli* strains to appropriately methylate plasmid DNA have been utilised by researchers to overcome this problem [5,13], however a universal and low-cost solution has not been developed. It is now well established that DNA methylation by RMS can influence bacterial gene expression and thus deletion of RMS is not an effective solution [14,16].

Phage have evolved a diverse array of strategies to counter bacterial RMS and facilitate infection, one example of which is the protein Ocr encoded by phage T7, a strongly negatively-charged DNA mimic that directly binds to and inhibits the activity of bacterial Type I RMS [17,18]. We recently developed a new protocol to improve the genetic tractability of bacteria expressing functional Type I RMS using a commercially produced recombinant Ocr protein [3] that has since been discontinued. Given the potential impact of Ocr on genetic manipulation of important pathogens, we have generated a new vector for expression of recombinant Ocr, and here describe a protocol for protein purification. We also assess recombinant protein activity demonstrating improvement in efficiency of DNA uptake to levels equivalent to those observed with the previously-available commercial product.

## Theory and implementation

**Fig. 1.**
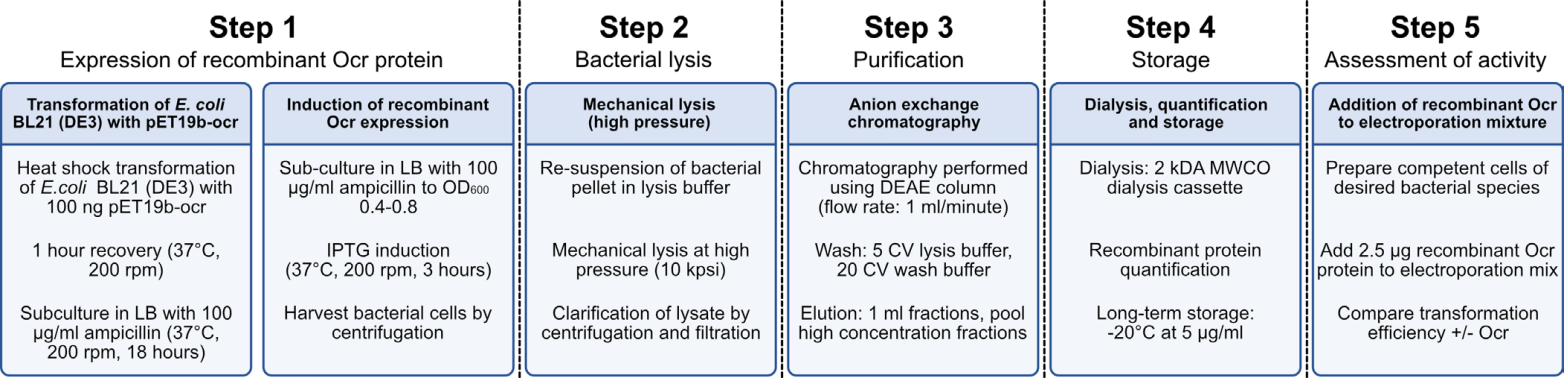
Schematic overview of methodology for expression and purification of recombinant Ocr protein. Detailed protocol described in the manuscript. CV=column volumes; MWCO=molecular weight cutoff.

Improving the efficiency of DNA uptake by bacterial pathogens is essential to facilitate the continued success of molecular microbiological research, with key impacts on determining the virulence mechanisms of important and clinically-relevant pathogens. Here, we present the method for in-house generation of a cost-effective and highly active recombinant phage protein that inhibits the activity of Type I RMS, with the potential to promote DNA uptake by a diverse array of genetically recalcitrant bacterial strains. Detailed methods are provided below, and are summarised in Fig. 1.

## Generation of expression vector pET19b-ocr

The expression vector pET19b-ocr was generated by ligating the *ocr* gene into the commercially available plasmid, pET19b (Novagen) that enables IPTG-inducible recombinant protein expression. Primers pET-ocr_F (5′-CATG**CCATGG**CTATGTCTAACATGACTTAC-3′) and pET-ocr_R (5′-CG**GGATCC**TTACTCTTCATCCTCCTCG-3′) were used to amplify the *ocr* gene from plasmid pAR2993 [19] incorporating the restriction sites NcoI and BamHI into the resulting PCR product to facilitate ligation into the vector pET19b. Digestion of the vector with NcoI resulted in removal of the sequence for a 6x His-tag from pET-19b-ocr, as the positive charge associated with this tag was anticipated to impair activity of the purified Ocr protein and reduce efficiency of anionic exchange chromatography for purification. The resulting vector was subjected to Oxford Nanopore sequencing (MicrobesNG) for confirmation and used to facilitate expression of recombinant Ocr protein.

## Step 1: expression of recombinant Ocr protein

### Transformation of *E. coli* BL21(DE3) (NEB) with pET19b-ocr plasmid DNA:

Recombinant Ocr protein was expressed in *E. coli* BL21(DE3) (NEB) following transformation with pET19b-ocr plasmid. Briefly, 2 µl plasmid DNA (at 50 ng µl^−1^) was incubated with *E. coli* BL21(DE3) on ice for 30 min, before being subjected to heat shock (42 °C, 10 s). The mixture was then placed on ice for 5 min, recovered in 1 ml SOC medium (37 °C, 200 r.p.m., 60 min) and 50 µl of the resulting culture was spiked into 50 ml lysogeny broth (LB) (100 µg ml^−1^ ampicillin) (37 °C, 200 r.p.m., 18 h).

### Induction of recombinant Ocr expression:

*E. coli* expressing pET19b-ocr were sub-cultured 1 : 100 in 1 litre of LB supplemented with ampicillin (100 µg ml^−1^) (37 °C, 200 r.p.m.) to an OD_600_ of 0.4–0.8. Protein expression was induced by the addition of IPTG (1 mM) and subsequently cultured for 3 h (37 °C, 200 r.p.m.). Bacterial cells were then harvested by centrifugation (4000***g***, 4 °C, 20 min,) and stored at −20 °C prior to protein purification.

## Step 2: bacterial lysis

Bacterial pellets were resuspended in lysis buffer (50 mM sodium phosphate, 50 mM ammonium chloride, pH8.0), and mechanically lysed by subjecting cells to high pressure (10 kpsi) (One Shot Cell Disruptor, Constant Systems). Mechanical lysis may also be performed using a sonicator [20]. The lysate was further clarified by centrifugation (4000***g***, 4 °C, 20 min), and the resulting supernatant filtered with a 0.2-μm-pore-size filter.

## Step 3: purification of recombinant Ocr protein

Ocr is a small (13.8 kDa) strongly negatively charged protein (theoretical pI: 3.87) that acts as a DNA mimic. As such, the inclusion of any tag to facilitate recombinant protein purification has the potential to impair activity and block function. A purification method based on charge, and performed using anion exchange chromatography was therefore developed [19,21, 22]. Purification was expedited using a Biorad EP-1 Econo Pump (flowrate: 1 ml min^−1^).

Bacterial lysates were applied to a HiTrap DEAE FF column (1 ml resin bed) (Cytiva), which had been pre-equilibrated with lysis buffer. The column was then washed with 5x CV of lysis buffer followed by 20 CV of wash buffer (50 mM sodium phosphate, 200 mM ammonium chloride, pH 8.0). Recombinant Ocr protein was then eluted in elution buffer (50 mM sodium phosphate, 600 mM ammonium chloride, pH8.0) in 1 ml fractions and quantified by UV absorption spectrophotometry using a Nanodrop (standard settings; 1 Abs=1 mg ml^−1^). Fractions containing >1 mg protein were pooled and taken forward.

## Step 4: preparation of recombinant Ocr protein for storage

The resulting protein was dialysed into dialysis buffer (100 mM Tris-HCl pH 7.5, 20 mM NaCl, 0.2 mM EDTA, 2 mM DTT) using a 2 kDa MWCO dialysis cassette (Thermo). Total protein was quantified by Bradford Assay (VWR) and diluted into the same storage buffer used for commercial Ocr protein (https://www.cambio.co.uk/289/10/products/typeone-restriction-enzyme-inhibitor/) (dialysis buffer with 0.1 % Triton X-100 and 50 % glycerol) to a final protein concentration of 5 µg µl^−1^. Protein aliquots were stored at −20 °C to avoid freeze-thaw cycles. SDS-PAGE was performed to validate the purity of the recombinant Ocr protein (Fig. S1). No reduction in activity following storage was observed (Fig. S2).

## Step 5: assessment of recombinant Ocr activity

Assessment of Ocr activity can be performed by addition of the recombinant protein to any experimental protocol for DNA uptake by bacteria, where an active Type I RMS is present. We have previously reported that *emm*1 group A streptococci exhibit high levels of genetic recalcitrance that limits the efficiency with which mutant strains can be generated. We went on to show that addition of a now discontinued commercial recombinant Ocr protein could improve the transformation efficiency by 1000-fold [3]. Here, we provide details of the modified protocol for transformation of group A *Streptococcus* with plasmid pDL278 [23] as an example of the impact recombinant Ocr can have on the genetic manipulation of a clinically significant pathogen, and to the validate the activity of our in-house produced recombinant Ocr protein.

## Transformation of group a *Streptococcus*

### Preparation of competent cells

Group A *Streptococcus* strain H584 (*emm*1) [24] was transformed with replicative plasmid pDL278 [23] as previously described [3]. Briefly, 40 ml bacteria were cultured in Todd Hewitt broth (37 °C, without shaking) to OD_600_ 0.2 and the cells harvested by centrifugation. The resulting pellet was washed five times in 1 ml ice-cold 0.5 M sucrose (16 000***g***, 1 min, 4 °C) and re-suspended in a final volume of 100 µl ice-cold 0.5 M sucrose. Then 50 µl aliquots of competent cells were added to pre-chilled tubes containing 5 µl of pDL278 plasmid DNA (100 ng µl^−1^), and 2.5 µg of recombinant Ocr (1 µl of a 1 : 2 dilution of stock protein) was added to one sample tube. All samples were stored on ice prior to electroporation.

### Electroporation

Bacterial cells were transformed by electroporation (MicroPulser Electroporator, Biorad) with the following settings: 200 Ω, 1.7 kV, 50 µF, 0.1 cm cuvette. Cells were recovered in 1 ml Todd Hewitt broth (37 °C, static, 1 h) and plated on Todd Hewitt agar supplemented with spectinomycin (50 µg ml^−1^) (37°C, 18 h).

## Step 6: confirmation of activity of in-house purified recombinant Ocr protein

**Fig. 2.**
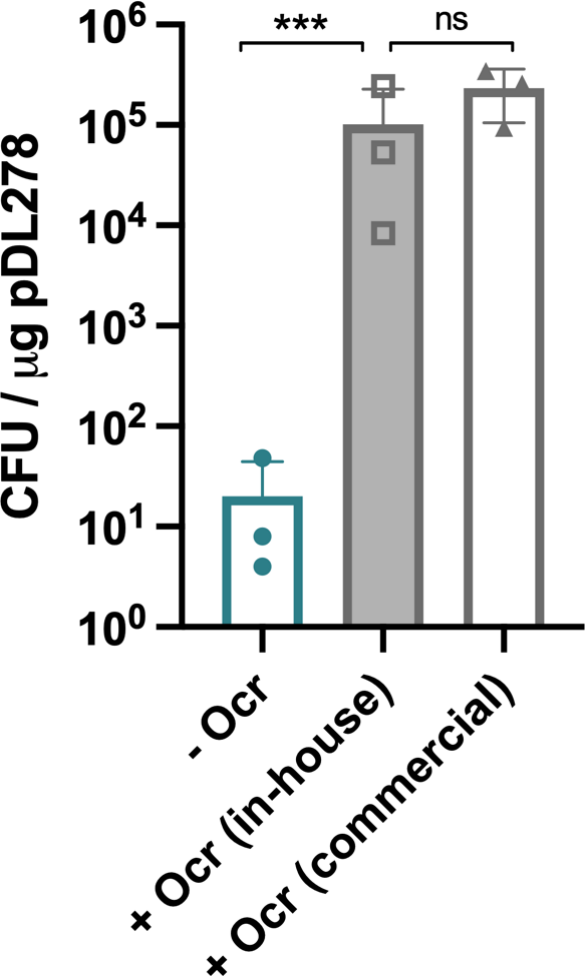
In-house purified recombinant Ocr exhibits equivalent activity to commercially produced Ocr. Quantification of the transformation efficiency of *emm*1 group A *Streptococcus* with DH5α-purified plasmid pDL278±recombinant Ocr (clear green bars = −Ocr; filled grey bars = +in-house purified Ocr; empty grey bars = +commercial Ocr). In-house purified recombinant Ocr improved transformation efficiency with plasmid pDL278 1000-fold, equivalent to that shown for commercially available protein. Data represent the mean and standard deviation of three independent experiments (ANOVA on log-transformed data; ***P<0.001).

Successfully transformed colonies were counted, and transformation efficiency calculated as bacterial cfu per microgram transformed plasmid DNA. In-house purified recombinant Ocr demonstrated equivalent activity to the commercially produced product (Fig. 2). Additional validation of the activity of in-house purified recombinant Ocr protein was performed using the *Salmonella enterica* Serovar Typhimurium strain LT2, transformation of which has previously been demonstrated to be enhanced by addition of Ocr protein [25]. Transformation efficiency of LT2 was significantly improved following addition of in-house purified Ocr protein (Fig. S3). Combined, these data validate the new tools generated and methodology provided here.

## Discussion

Genetic manipulation of clinically important bacterial species is critical to our continued understanding of emerging pandemic threats, as well as the alarming rise of antimicrobial resistance. Despite this, research into certain species and lineages is hampered by a lack of tools to overcome the naturally-occurring resistance mechanisms employed by bacteria to protect themselves from the acquisition of exogenous DNA [3,4]. The development of widely applicable and cost-effective methods that block these pathways are thus essential to facilitate fundamental research into all aspects of bacterial physiology.

RMS are expressed by over 90 % of bacterial strains that have been sequenced to date [6,26], and represent a universal barrier to uptake of exogenous DNA that limits the potential for genetic manipulation of many bacterial strains [27]. Type I RMS are present in 29.5 % strains, and are frequently associated with genetic recalcitrance [27]. Here we report the methods to express, purify and use a recombinant phage protein, Ocr, to inhibit Type I RMS, and provide proof-of-principle data for this highly efficient and cost-effective method for improving bacterial genetic tractability.

Capitalising on natural RMS inhibition strategies employed by phage represents a largely un-tapped reservoir of proteins that could be used to block the activity of different classes of RMS, and fully alleviate the negative impact of these systems on the genetic manipulation of diverse bacterial species. By developing protein-based inhibition systems the blockade on activity will be transient, and while sufficient to facilitate DNA uptake, will not impact subsequent activity of the RMS or modify the behaviour of the bacteria in any way. Such an approach represents a universal strategy to expand the diversity of bacterial strains that can be successfully subjected to genetic manipulation.

## supplementary material

10.1099/mic.0.001465 Uncited Supplementary Material 1.

## Abbreviations

ANOVA: analysis of variance
CV: column volume
GAS: group A *Streptococcus*
LB: lysogeny broth
MWCO: molecular weight cutoff
RMS: restriction modification system
