# Supplementary material for: Generation of tools for expression and purification of the phage-encoded Type I restriction enzyme inhibitor, Ocr
Source: Microbiology (Reading). 2024 Jul 23;170(7):001465. doi: 10.1099/mic.0.001465 (PMC11317969; doi:10.1099/mic.0.001465)
Supplement: Uncited Supplementary Material 1. [file mic-170-01465-s001.pdf]

Supplementary methods:

Transformation of *Salmonella enterica*

Preparation of competent cells:

Salmonella enterica Serovar Typhimurium strain LT2 was transformed with replicative plasmid pUC19 (NEB) as previously described (25). Briefly, 100 ml bacteria were cultured in LB (37°C, 180 rpm) to OD_{600} 0.6 and the cells harvested by centrifugation. The resulting pellet was washed 2x in 100 ml ice-cold distilled water and once in 20 ml ice-cold 10% glycerol (4000 xg, 10 minutes, 4°C), before being re-suspended in 200 µl ice-cold 10% glycerol. 40 µl aliquots of competent cells were added to pre-chilled tubes containing 1 µl of pUC19 plasmid DNA (1 µg/µl) +/- 2.5 µg recombinant Ocr. All samples were stored on ice prior to electroporation.

Electroporation:

Bacterial cells were transformed by electroporation (MicroPulser Electroporator, Biorad) with the following settings: 200 Ω, 2.5 kV, 25 µF, 0.2 cm cuvette. Cells were recovered in 1 ml pre-warmed SOC medium (37°C, 180 rpm, 1 hour), and plated on MacConkey agar supplemented with Ampicillin (100 µg/ml) (37°C, 18 hours).

Supplementary figures:

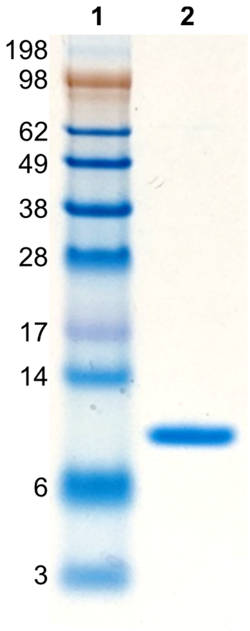

Supplementary Figure 1: Purity of recombinantly expressed Ocr protein. SDS-PAGE of in-house purified recombinant Ocr protein (1 µg). Ocr runs at expected size of 13.8 kDa. (Lane 1 = molecular weight marker; lane 2 = recombinant Ocr protein (1 µg).

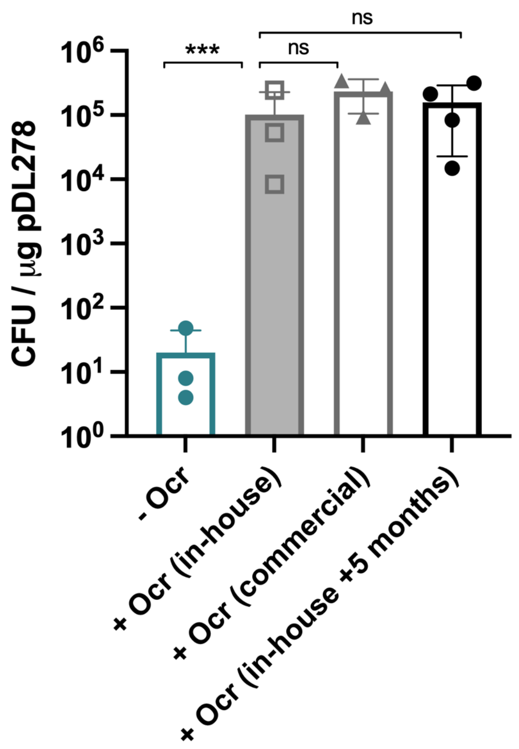

Supplementary Figure 2: In-house purified recombinant Ocr stored for 5 months at -20°C exhibits equivalent activity to freshly purified recombinant protein. Quantification of the transformation efficiency of *emm*1 group A *Streptococcus* with DH5α-purified plasmid pDL278 ± recombinant Ocr (clear green bars = −Ocr; filled gray bars = +in-house purified Ocr; empty gray bars = +commercial Ocr; clear black bars = +in-house purified Ocr stored for 5 months at -20°C). Stored protein activity was compared to that of freshly purified protein using the original transformation efficiency data included in Figure 2 of the manuscript. In-house purified recombinant Ocr stored at -20°C for 5 months exhibited a transformation efficiency equivalent to that shown for freshly purified recombinant protein. Data represent the mean and standard deviation of three independent experiments (ANOVA on log-transformed data; *** $p < 0.001$, ns: $p > 0.05$).

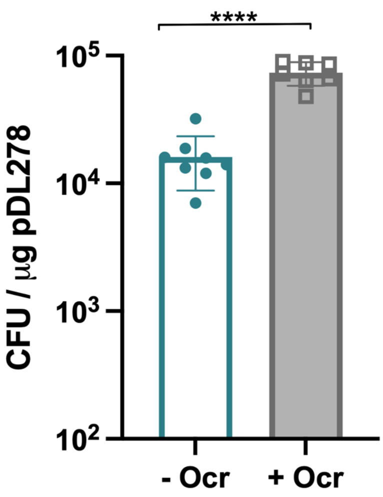

Supplementary Figure 3: Transformation of *Salmonella enterica* Serovar Typhimurium strain LT2 is enhanced following addition of recombinant Ocr protein. Quantification of the transformation efficiency of *Salmonella enterica* Serovar Typhimurium strain LT2 with DH5α-purified plasmid pUC19 ± recombinant Ocr (clear green bars = −Ocr; filled gray bars = +in-house purified Ocr). Data represent the mean and standard deviation of 7 or 8 independent experiments (Student's T test; **** $p < 0.0001$).
